# Figure–ground discrimination behavior in *Drosophila*. I. Spatial organization of wing-steering responses

**DOI:** 10.1242/jeb.097220

**Published:** 2014-02-15

**Authors:** Jessica L. Fox, Jacob W. Aptekar, Nadezhda M. Zolotova, Patrick A. Shoemaker, Mark A. Frye

**Affiliations:** 1 Howard Hughes Medical Institute and Department of Integrative Biology and Physiology, University of California Los Angeles, Los Angeles, CA 90095-7239, USA; 2 Tanner Research, Monrovia, CA 91016, USA

**Keywords:** Fly vision, Flight control, Figure tracking, Optomotor response

## Abstract

The behavioral algorithms and neural subsystems for visual figure–ground discrimination are not sufficiently described in any model system. The fly visual system shares structural and functional similarity with that of vertebrates and, like vertebrates, flies robustly track visual figures in the face of ground motion. This computation is crucial for animals that pursue salient objects under the high performance requirements imposed by flight behavior. Flies smoothly track small objects and use wide-field optic flow to maintain flight-stabilizing optomotor reflexes. The spatial and temporal properties of visual figure tracking and wide-field stabilization have been characterized in flies, but how the two systems interact spatially to allow flies to actively track figures against a moving ground has not. We took a systems identification approach in flying *Drosophila* and measured wing-steering responses to velocity impulses of figure and ground motion independently. We constructed a spatiotemporal action field (STAF) – the behavioral analog of a spatiotemporal receptive field – revealing how the behavioral impulse responses to figure tracking and concurrent ground stabilization vary for figure motion centered at each location across the visual azimuth. The figure tracking and ground stabilization STAFs show distinct spatial tuning and temporal dynamics, confirming the independence of the two systems. When the figure tracking system is activated by a narrow vertical bar moving within the frontal field of view, ground motion is essentially ignored despite comprising over 90% of the total visual input.

## INTRODUCTION

A natural visual scene contains stimuli of varying salience and relevance, and animal visual systems must segregate these stimuli for appropriate behavioral responses. During locomotion, the task of discriminating features is complicated by self-generated ground motion. Optokinetic responses (Hassenstein and Reichardt, 1956; Götz and Wenking, 1973) help by adjusting the animal's gaze to stabilize wide-field ground motion. However, how do animals manage the optokinetic response while simultaneously tracking a figure that may be ethologically relevant, but composed of visual cues that are substantially smaller and weaker than the ground?

The fly visual system shares many structural and functional traits with that of vertebrates (Sanes and Zipursky, 2010). Flies readily pursue salient features while walking (Horn and Wehner, 1975; Robie et al., 2010) and in flight (Land and Collett, 1974), and figure fixation in tethered flies can be evoked with a simple contrasting vertical bar (Kennedy, 1940; Götz, 1975; Reichardt and Poggio, 1976; Bahl et al., 2013). Additionally, flies correct wide-field ground motion perturbations in a manner analogous to human optokinetic responses (Hassenstein and Reichardt, 1956; Götz and Wenking, 1973; Götz, 1975; Paulus et al., 1984; Lappe et al., 1999). Figure and ground responses are distinguished from one another by their sensitivity to stimulus size, interocular properties and dynamics (Egelhaaf et al., 1988; Kimmerle et al., 1997; Duistermars et al., 2007). Furthermore, whereas ground responses are, by construction, uniform with respect to image position, figure tracking is tuned such that sensitivity is maximal when the figure is located in the frontal field (Reichardt and Poggio, 1976; Aptekar et al., 2012). Less is known, however, about how figure and ground systems interact when both signals are present, which is normally the case in flight. How might figure and ground systems interact, and how might that interaction vary in space and time?

To examine figure-tracking behavior with the classical optomotor ground response, we measured the wing-steering behavior of tethered fruit flies (*Drosophila melanogaster* Meigen 1830) while they were shown a compound visual stimulus consisting of a moving 30 deg figure against a moving panorama presented simultaneously. We measured the correlation between their wing-steering behavior and the motions of both the moving figure and the moving ground to quantify the strength of the fly's behavioral response to each stimulus component. In doing so, we constructed spatiotemporal action fields (STAFs) (Aptekar et al., 2012), which are analogous to the spatiotemporal receptive fields (STRFs) used to describe sensory systems, but which measure the behavioral response of an animal instead of the membrane voltage response of one or more neurons. By measuring STAFs for simultaneous figure and ground motion, we find that responses to figures and wide-field ground motion are spatially and temporally distinct, and that the total steering effort is shared between the two responses. Most notably, the presence of a figure in the frontal field of view strongly suppresses wide-field ground optomotor responses. This suppression does not result simply from decreased wide-field input resulting from masking by the figure, but rather the figure system actively suppressing the optomotor reflex to enable figure pursuit independently from corruption by wide-field ground input.

## RESULTS

### Terminology

**Fig. 1.**
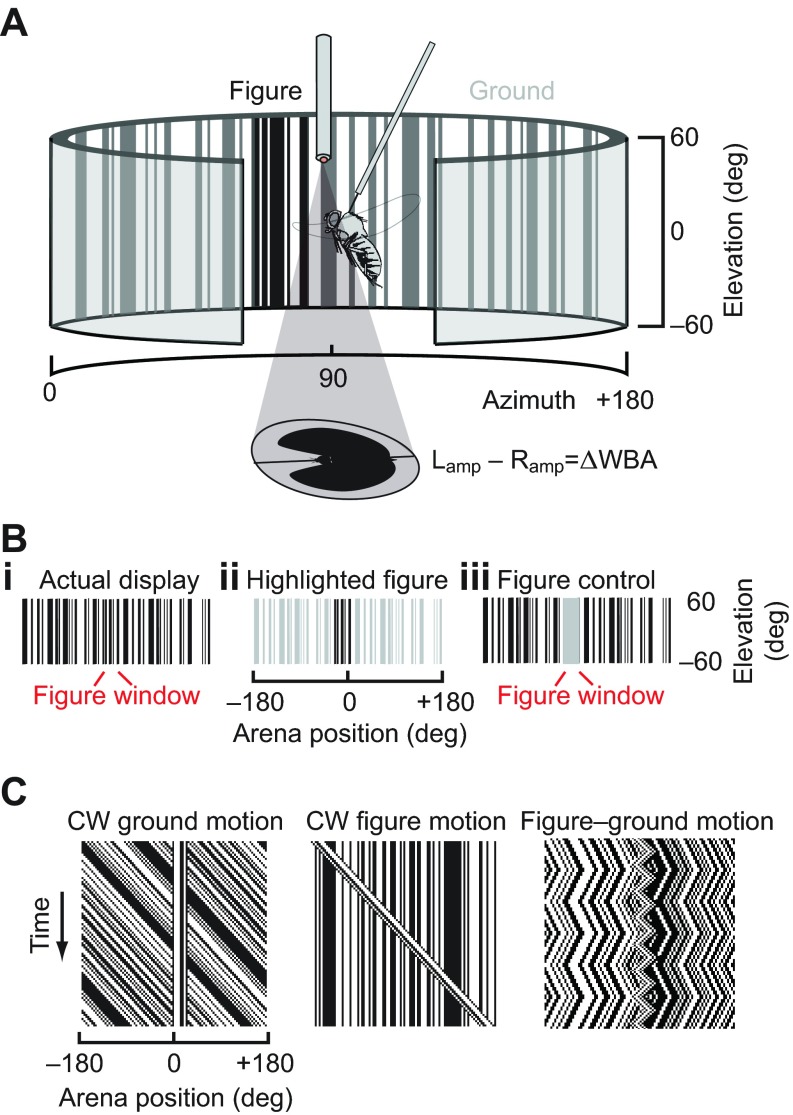
**Experimental setup.** (A) A fly is suspended within a circular LED visual display. Steering responses are monitored with a photodiode system measuring the amplitude of each left and right wingbeat, the difference of which (ΔWBA) reflects steering torque. (B) One-dimensional random period vertical gratings create the visual ground (i) and 30 deg figure (ii), such that the figure is defined only by its relative motion. Control experiments use a sparse dot pattern (iii) and one with a 30 deg ‘window’ of isoluminant uniform gray designed to reduce the amount of correlated ground motion signal as in the experiment shown in Bi, but without providing relative figure motion signals. (C) Space–time graphs illustrating a fixed figure with clockwise (CW) ground motion, fixed ground with clockwise figure motion and a figure oscillating on a ground with distinct dynamics.

A ‘figure’ is a closed area of visual space wherein the spatiotemporal statistics of motion are distinct from the background. The figure we use here is a ‘Fourier bar’, a solid object in which the boundaries of the figure and the texture that lies between those boundaries (i.e. the surface of the object) always move together. A Fourier bar evokes a response to both its ‘elementary motion’ (EM) – the velocity of the textured surface – and to its ‘figure motion’ (FM) – the moving position of the figure boundaries (Zanker, 1993; Theobald et al., 2008; Aptekar et al., 2012). The Fourier bar is considered a ‘small-field’ stimulus. By contrast, the rest of the visual panorama moving coherently is considered a ‘wide-field’ stimulus. For brevity, we refer to the Fourier bar simply as the ‘figure’ and the wide-field panorama as the ‘ground’ (Fig. 1), following prior convention (Reichardt and Poggio, 1979). It should be noted that the figure here is defined only by its motion relative to the ground – if always moved synchronously with the ground, the figure, which is itself a vertical patch of a random pattern, cannot be distinguished from the background by any measure (Fig. 1Bi). However, if held stationary on a moving ground, or if moved in an uncorrelated way from the ground, the figure boundaries are easily identified as seams in the EM flow field composing the ground (Fig. 1C).

### In flight, flies actively fixate a figure against a moving ground, even when figure and ground motion directly oppose

**Fig. 2.**
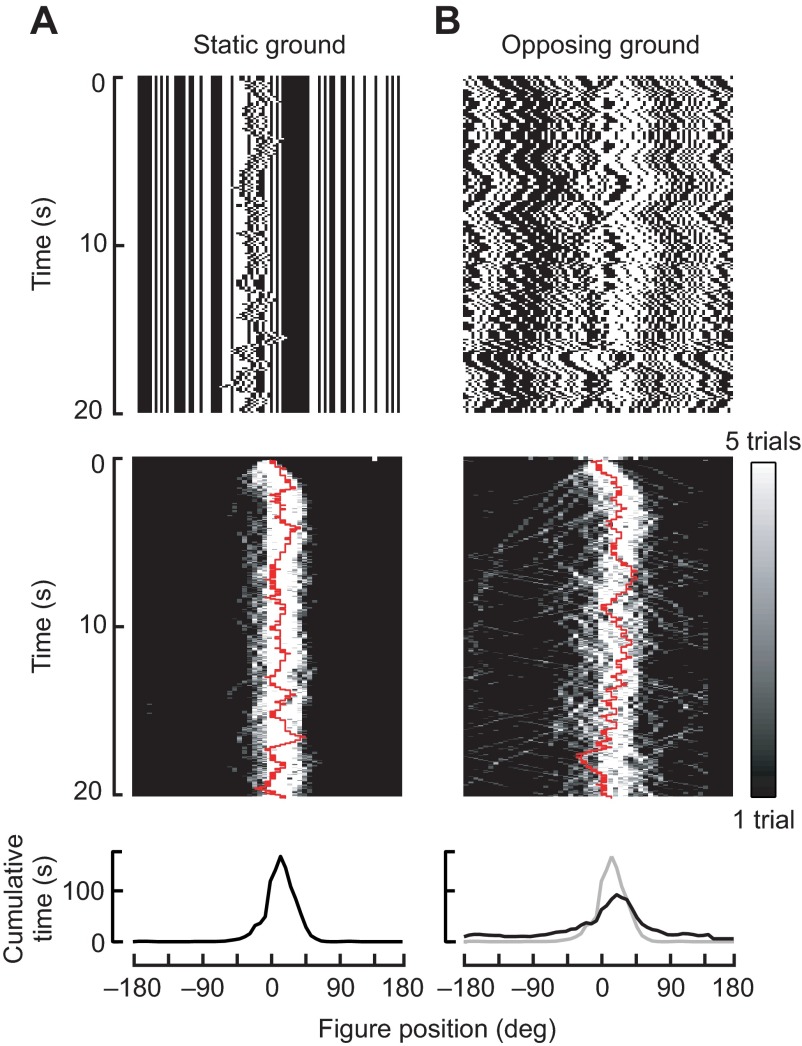
**Closed-loop figure fixation on a static ground (A) or on a ground that moves opposite figure motion (B).** Top row: space–time graphs of visual stimuli generated by an experimental trial. Middle row: time histograms of bar position, actively controlled by a feedback loop coupling ΔWBA to figure velocity of the figure. Grayscale represents the number of trials in which a fly held the bar at each position over time (e.g. black indicates that no flies fixated the bar at a particular point in space and time; white indicates that many flies fixated the bar). The red line shows one sample trace, which generated the image motion plotted in the top row. Bottom row: histograms of bar position during the last 10 s of each trial. (A) The bar is under closed-loop control against a static ground of identical texture. (B) The closed-loop bar is set against a counter-rotating ground such that any steering increment that moves the bar to the left moves the ground to the right. Gray line shows the histogram for the static-ground experiment for comparison. *N*=16 flies.

In closed-loop experiments in which flies are allowed to control the position of a figure against a static background, we found they use relative motion signals to robustly fixate the figure on the visual midline when it is set against a static ground (vector strength=0.93, *P*<0.05; Fig. 2A). In the experiments in which the background moves and is inversely coupled to figure motion, we found that although the stimulus generates a noisier spatial distribution (Fig. 2B, top), flies nevertheless tend to fixate the bar in the frontal field of view (Fig. 2B, bottom). Figure tracking performance is slightly degraded by comparison to the static ground condition (vector strength=0.61, *P*<0.05), but flies nonetheless show a statistically significant ability to fixate the figure despite directionally opposing motion stimuli that occupy 92% of their visual field, and which ought to be generating directly opposing wide-field optomotor responses.

### STAFs for wide-field ground motion and figure fixation show distinct spatial tuning and dynamics of steering responses

**Fig. 3.**
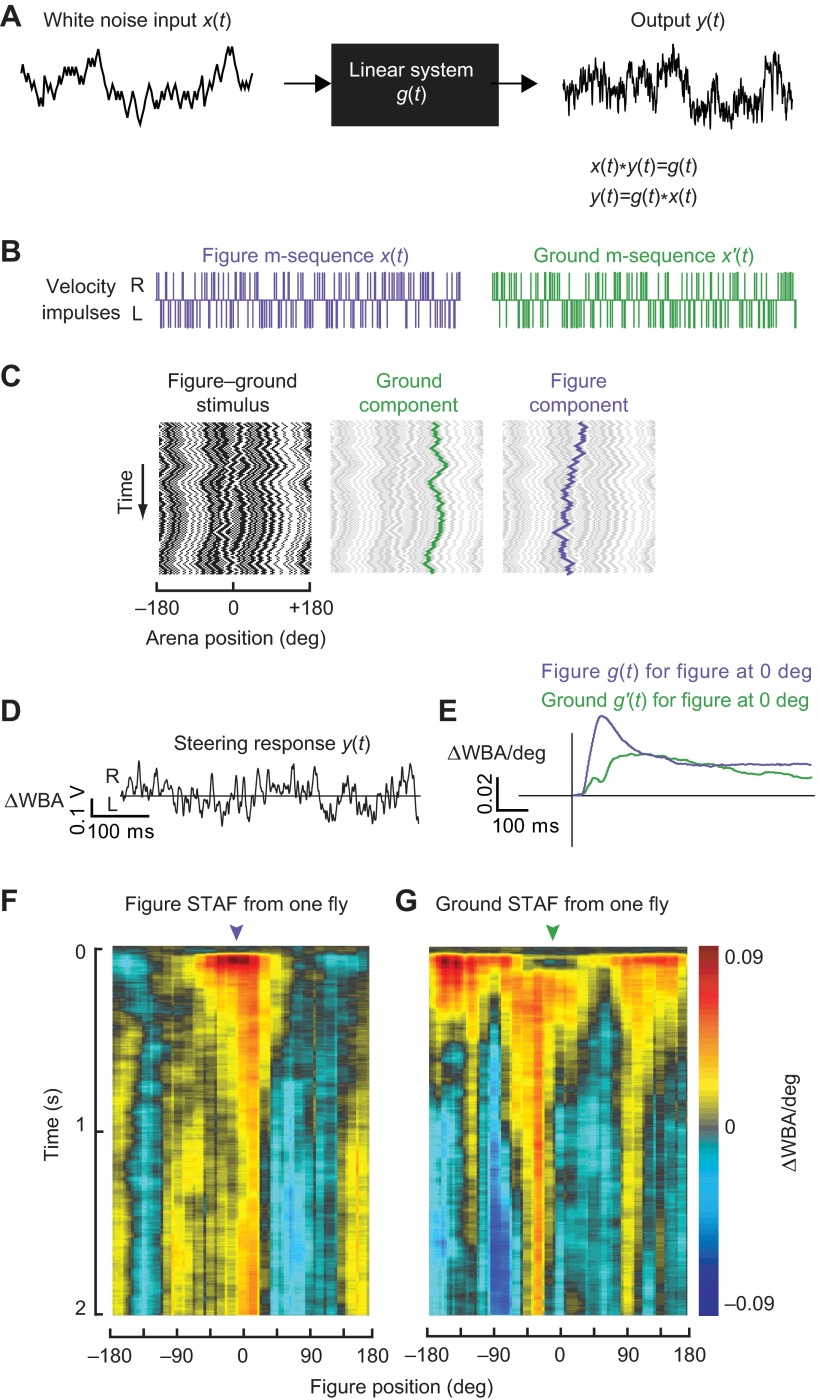
**Cross-correlation method for linear filter calculation.** (A) Schematic of linear input–output system. (B) Independent maximum length sequences (m-sequences) drive figure and ground pattern motion independently. Individual velocity impulses drive the pattern one display pixel (3.75 deg) to the right (R) or the left (L). (C) Compound stimulus in which the figure and ground patterns are controlled by independent m-sequences and superposed on the arena display. The ground component is highlighted in green, the figure component in purple. (D) The fly's steering response to the compound figure–ground stimulus as measured by the difference between left and right wingbeat amplitudes (ΔWBA, in V; uncalibrated yet directly proportional to angular wingbeat amplitude). (E) Linear filter kernels for figure motion and ground motion as calculated by cross-correlation of the steering response with the figure or ground motion, respectively. (F) The figure spatiotemporal action field (STAF) measured from a single fly. (G) Same as F for the ground STAF measured simultaneously from the same fly.

We aimed to characterize the spatiotemporal properties of optomotor responses to both wide-field ground motion and figure motion for varying azimuthal positions of the figure. Adapting previous methodology (Theobald et al., 2010a; Chow et al., 2011; Aptekar et al., 2012), we treated the fly as a linear transducer of visual motion into steering wing kinematics (Fig. 3A). Note that this does not imply that the underlying neural, musculoskeletal or biomechanical events connecting visual stimulation with steering behavior are themselves linear, but rather that the sensorimotor cascade that transforms figure and background motion into motor responses can be approximated by linear operators over sufficiently restricted ranges of figure composition, position and speed, and background image speed.

We programmed the figure to move along one maximum length sequence (m-sequence), and the randomly patterned ground to move along a second, independent m-sequence (Fig. 3B). The resulting apparent motion signal is then a series of steps in image position, corresponding to impulses in image velocity of both the ground and

figure components (Fig. 3C). The steering response (i.e. the difference in amplitude between the left and right wings, ΔWBA) produced by the fly in response to this compound visual stimulus (Fig. 3D) was processed by circular cross-correlation with each of the figure and ground m-sequences over a sliding window of 127 samples (Aptekar et al., 2012). Because of the auto- and cross-correlation properties, these yield independent estimates of the figure and ground kernel functions, respectively, from the same set of data. The cross-correlation with the ground sequence was performed directly with the ΔWBA data; however, because the figure can produce persistent responses when off the midline, the cross-correlation with the figure sequence was performed with the time derivative of ΔWBA and the result subsequently integrated to obtain the kernel function, in order to preclude foldback of responses from prior cycles of the m-sequence into the cross-correlation (Aptekar et al., 2012). Numerical measures were also taken to correct for a small DC error term in the kernel estimate that is inherent to the m-sequence method (Aptekar et al., 2012). The resulting impulse response estimates were divided by the magnitude of image displacement at each element of the m-sequence (3.75 deg) to give velocity impulse response estimates with dimension ΔWBA(V)/deg.

**Fig. 4.**
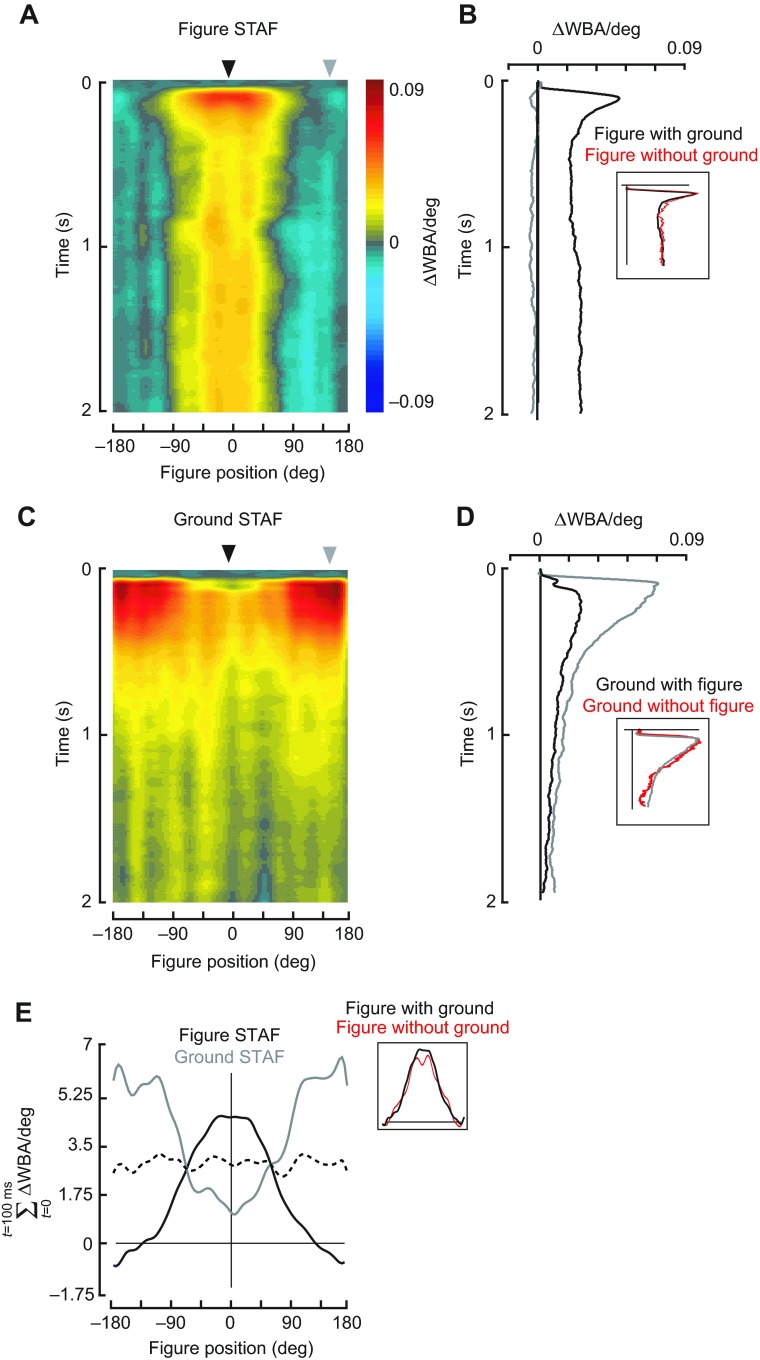
**Figure–ground discrimination STAFs constructed for a stimulus in which a randomly striped figure moved against a randomly striped ground on separate m-sequences.** (A) For compound figure–ground stimuli, wing optomotor steering responses correlated with the movement of the figure. Impulse responses are parameterized to the azimuthal position of the bar (e.g. responses appearing on the midline are calculated when the bar is directly in front of the fly). (B) The figure response measured with the figure in the front (black) and rear (gray) of the arena, indicated by the arrowheads in A. Inset: figure responses compared with and without concomitant ground motion [red line; re-plotted from Aptekar et al. (Aptekar et al., 2012)]. (C) For compound figure–ground stimuli, wing optomotor steering responses correlated with the motion of the wide-field ground parametized by the azimuthal position of the bar. (D) The ground response measured with the figure in the front (black) and rear (gray) of the arena from the positions indicated by the arrowheads in C. Inset: ground responses re-plotted from D (gray) for the random striped pattern compared with a yaw pattern that contains no figure [red; re-plotted from Theobald et al. (Theobald et al., 2010a) on the same time scale]. (E) Azimuthal profile of the STAFs summed over the first 100 ms, highlighting the spatial variation of the impulse response amplitudes. Black line, figure; gray line, wide-field ground; dashed black line, mean of figure and ground. Inset: figure STAF spatial profiles compared for concomitant ground motion (E) and a stationary ground [red line; re-plotted from Aptekar et al. (Aptekar et al., 2012)]. *N*=27 flies.

We reasoned that the decreased tracking fidelity during the counter-rotating closed-loop stimulus (Fig. 2) is due to sharing of the control effort between wide-field ground stabilization and figure tracking. This motivated our efforts to measure responses to figure and ground motion simultaneously. We constructed STAFs by moving the figure and the ground on independent white noise sequences and measuring the wing steering impulse response to figure motion and ground motion for discrete horizontal positions of the figure on the visual arena. We found that the impulse response filters for the figure are spatially and temporally different from the filters for the ground, parameterized by figure location (Fig. 4). It is apparent that the figure responses build to their largest amplitudes within an area centered in the frontal field of view, and fall off in the periphery, such that figures appearing in the rear half of the field of view generate decreased tracking responses (blue areas in Fig. 4A). The figure response is nearly identical to that measured against a static ground by Aptekar et al. (Aptekar et al., 2012) (Fig. 4B, inset).

In the same experiment, the steering effort correlated with motion of the wide-field panorama (itself uncorrelated with the figure motion) generates a ground STAF indicating robust responses to panoramic motion only when the figure is outside the frontal field of view. That is, when the figure appears in the frontal field, steering responses are only weakly correlated with ground motion. When the figure moves through the periphery into the rear field of view, figure-tracking efforts are reduced and the effect of background motion becomes more significant (Fig. 4C,D). Spatial profiles of the STAFs summed over the first 100 ms of the response show that the maximal figure response occurs in the region of space where the ground response is minimal, and vice versa (Fig. 4E). It bears repeating that because the STAFs represent the incremental steering response over the azimuth, the regions of the STAF spatial profile with the largest magnitudes represent the retinal locations where the fly's response to any incremental change in figure displacement elicits the maximal change in steering response.

### The presence of a figure is required for active suppression of the wide-field stabilization response

**Fig. 5.**
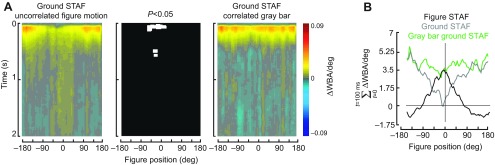
**Control STAF in which the uncorrelated figure has been replaced with an equal-sized uniform gray patch embedded in the ground pattern.** STAFs were measured in the same manner as in Fig. 4. (A) Left: ground STAF parameterized for the azimuthal position of a 30 deg figure moving on a separate m-sequence. Note the prominent response suppression on visual midline. Right: ground STAF in which a 30 deg region of the random black-and-white pattern has been replaced with a gray patch of equal mean luminance (Fig. 1Biii). The center plot shows the regions of significant difference between the two ground STAFs. White indicates statistical significance (*P*<0.05, paired *t*-test after Benjamini–Hochberg false discovery rate correction). (B) Azimuthal profile of the STAFs summed over the first 100 ms.

The apparent ‘notch’ in the center of the ground STAF on the visual midline (Fig. 4) would indicate that active figure tracking near the midline results in active inhibition or suppression of the ground optomotor response. An alternative explanation, however, is that the sensitivity of the ground system is anisotropic, weighted near the visual midline, and that occluding the coherent ground motion over the frontal visual field explains the apparent ground suppression effect. In the experiments in which an equiluminant gray bar replaced the figure and moved with the background, we found that its presence did not suppress the ground response when it was centered on the fly's visual midline (Fig. 5), and that the ground STAFs differed only in this regard: statistical comparison of the two STAFs shows that the only area of difference in space and time is the small region that describes the early response at the midline of the visual field (Fig. 5A, middle).

### Size tuning of the figure and wide-field STAFs

**Fig. 6.**
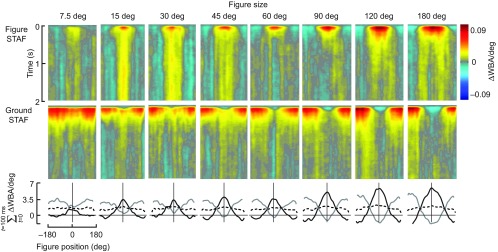
**Influence of varying figure size on figure and ground STAFs.** STAFs are compiled as in Fig. 4, but for figures of varying size as identified. Bottom row: azimuthal spatial profiles of the figure STAF (black) and ground STAF (gray). *N*=24 flies for 7.5, 15, 45, 60, 90 and 120 deg STAFs; *N*=18 flies for the 30 and 180 deg widths.

To examine the spatial limitations of the figure and wide-field systems, we systematically varied the size of the figure and constructed STAFs for figures of 7.5, 15, 30, 45, 60, 90, 120 and 180 deg. First, we note that the amplitude of the ground response increases as the figure size increases (Fig. 6). If the spatial structure of the ground STAF, with reduced amplitude and temporal delay when the figure is frontal, were simply due to a masking of the ground stimulus by the figure, then we would expect the overall amplitude of the ground STAF to be diminished by an increasingly large figure. That the data show the opposite is a second indication that the interaction of the figure and ground systems cannot be an artifact of masking the ground system, but rather must be due to a mechanism engaged by figure tracking, consistent with our control experiments (Fig. 5).

But what explains the peculiar increase in STAF amplitude with increasing figure size, particularly for the figures 60 deg in width and greater (Fig. 6)? The ground pattern generates a pattern of rotational yaw optic flow, and when the embedded figure is narrow, this rotation is uniform about the yaw axis across most of the visual field. However, as the figure increases in size, the pattern of internal optic flow may be ‘interpreted’ by the optomotor stabilization system as local wide-field motion in whatever direction the figure is moving. Prior work has demonstrated that a yaw flow field that has been directionally inverted across the rear hemisphere, producing a pattern of side-slip, generates optomotor responses several fold larger than those of yaw stimuli (Tammero et al., 2004; Duistermars et al., 2007; Theobald et al., 2010a). Increasing the size of the figure to 180 deg results in front and rear hemisphere motion that may more closely resemble slip optic flow than a figure moving on the ground. Slip responses are much larger than yaw, which by our reasoning explains the systematic increase in the STAF amplitudes. More work is required to test this hypothesis.

### Temporal properties of the STAFs

**Fig. 7.**
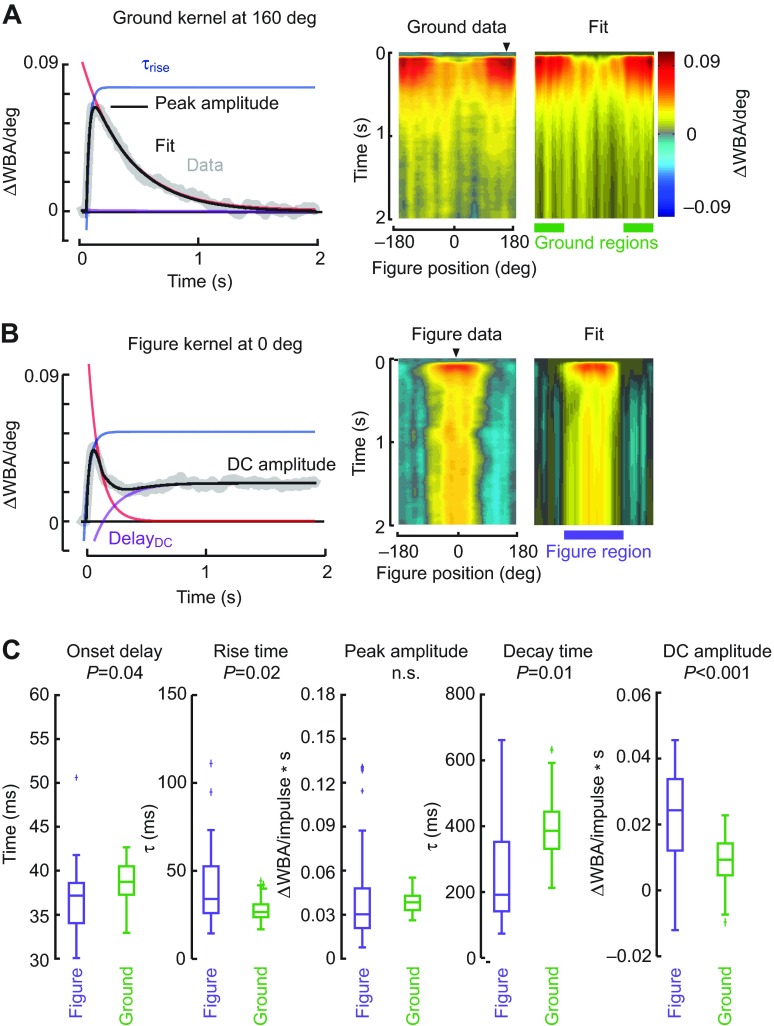
**Analysis of STAF temporal dynamics.** (A) Left: The ground kernel measured at 157 deg was fit with a model based on the difference of two exponentials (see Materials and methods for details). Right: the fitted ground responses at each azimuthal location are compiled within a heat map in the same manner as the data STAF to enable visual comparison. Arrowheads on the right-hand panels indicate the azimuthal location of the bar for the data plotted in the left-hand panels. (B) Fits for the figure response measured at 0 deg (left), and for all azimuthal positions complied into a STAF (right). (C) Parameter values are compared between the figure and ground responses for kernels occurring where the figure responses were strongest (at 0 deg azimuth, indicated by arrowhead in B) and where the ground responses were strongest (at 157 deg azimuth, indicated by the arrowhead in A), respectively. Box-and-whisker plots indicate the median or second quartile, first and third quartiles (box), and 1.5 times the upper and lower quartile range (whiskers). Outliers are marked with plus signs. *P*-values are reported for the unpaired *t*-test and Bonferroni correction. The spatial regions of maximum ground response and maximum figure response, from which the parameters are drawn spatially, are indicated with green and purple bars, respectively.

Each of the impulse response filters reflects properties of the fly's behavioral response to a velocity impulse, and our fits to the kernel functions parameterize them by onset delay between the visual stimulus and steering response, rise time, peak amplitude, decay time and amplitude of the DC position response. Fitting the kernels collected at each azimuthal position demonstrates how well the variation in temporal parameters agrees with the measured data (Fig. 7). Similarly, for figure responses measured with the figure at 0 deg azimuth, the fitted parameters at that spatial location compare well with the measured data, and thus the fitted STAF is nearly indistinguishable in space and time from the measured one (Fig. 7B).

The very close agreement between the individual kernels (Fig. 7A) and the fully compiled STAFs allows us to quantify how the key temporal response parameters vary between the figure and

ground STAFs. We note the following similarities and differences. First, for their respective spatial regions of sensitivity, peak amplitudes of the figure and ground STAFs are very similar. This is notable because the figure constitutes <10% of the visual input to the fly at any point in the visual field, and argues for substantial amplification of motion cues contained within a figure relative to the ground, at least for the experimental conditions used in the present study. Second, while the difference in the rise time constants of the two systems is statistically significant, they differ by <10 ms, whereas the decay time constants differ by more than 200 ms. This means that the two systems have substantively similar frequency responses for rapidly varying stimuli – greater than ~20 Hz – but

diverge only for more slowly varying stimuli. This finding agrees with frequency domain analyses using periodic stimuli (Duistermars et al., 2007). Finally, the DC position response is substantially larger in the figure system relative to the ground system. This reflects the fact that figure tracking has an important positional (DC) component, whereas ground stabilization can be accomplished, similar to EM tracking, with a nearly pure velocity-nulling control algorithm. However, a velocity-only system is ill-suited to figure tracking, because it is susceptible to low-frequency drift away from the target being tracked. This drift is compensated by the DC component of the figure control system. Overall, the dynamics of figure tracking and ground stabilization are clearly distinct (Fig. 7C), supporting prior conclusions that the behaviors are served by independent pathways (Egelhaaf et al., 1988). Further, this analytical fitting strategy can help anchor a control theoretic framework for either implementing an autonomous control system or studying closed-loop control (Shoemaker et al., 2011).

### STAFs predict fly responses to figures moving against a background

**Fig. 8.**
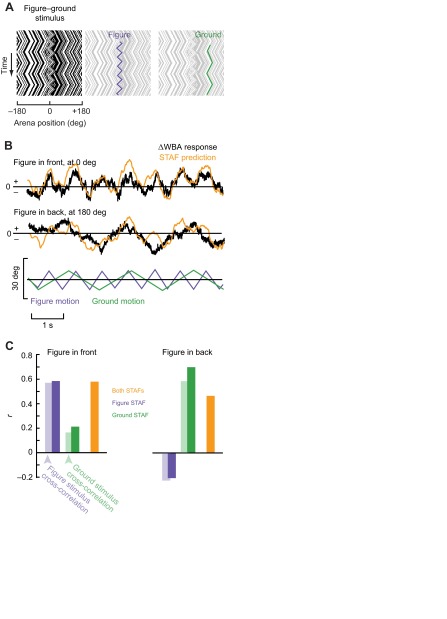
**Measuring and modeling responses to figures moving against a moving background.** (A) The figure and the ground were oscillated according to two constant-velocity triangle-wave position trajectories. (B) Average ΔWBA responses (black) when the figure was oscillated in front of or in back of the flight arena. Predictions made by convolving the combined figure STAF and ground STAF (Fig. 3) with the compound triangle-wave stimulus (orange). (C) Correlations between the figure or ground motion and the measured response (transparent bars); and correlations between the predictions made with the figure STAF, the ground STAF or both, and the measured response (solid bars). *N*=10 flies, 5 trials each.

STAFs enable rapid visual inspection of the spatio-temporal properties of visuo-motor integration at the behavioral level, but

they also represent linear filters that provide the means to predict responses to input signals. We therefore examined how well the STAFs, independently and in combination, could predict the fly's steering responses to simple periodic stimuli. To do this, we oscillated the ground and figure with constant-velocity triangle-wave position trajectories at two different frequencies and measured the flies' open-loop steering responses (Fig. 8A). Because the share of total steering effort dedicated to figure tracking (relative to wide-field stabilization) is largest when the figure is located in the front and smallest when the figure is in the rear visual field, we tested the flies with the figure stimulus centered in either the front or back of the arena.

We examined the predictive power of the figure and ground STAFs by spatially convolving them with the triangle-wave stimuli and comparing the result with the measured fly behavioral responses to the same signals (Fig. 8B). In addition, by convolving the figure and background kernels separately, we were able to test the potential for each subsystem to predict the response. We found that when the bar was oscillated in front of the fly, the figure response comprised the majority of the fly's behavior and the ground response contributed much less, reflected in the respective *r*-values (Fig. 8C). By contrast, when the figure was oscillated in the rear of the visual arena, the prediction made by the figure component of the STAF was slightly negatively correlated with the fly's steering effort, and the ground component of the STAF explained a far greater amount of the variance (Fig. 8B). The combined STAFs are able to account for a large part of the variance in the fly's turning effort in all simulations (*r*=0.46–0.58).

Additionally, we determined the correlation coefficient between the two stimulus component motions and the steering response to quantify how much of the flies' behaviors could be explained by each of the two stimulus components (Fig. 8C, light-shaded bars). Consistent with the spatial variation in the figure STAF (Fig. 4), when the figure is in front of the fly, the figure motion explains a large portion of the variance (*r*=0.57) while the ground motion is far less correlated with the response (*r*=0.17; Fig. 8C, left light-shaded bars). However, when the figure is oscillated behind the fly, the figure motion is markedly less correlated with the response (*r*=−0.22) and the ground component

of motion explains the majority of the flies' responses (*r*=0.58; Fig. 8C, right light-shaded bars).

We calculated the *r*-value for the correlation between the ΔWBA for each of our 50 trials in pairs. The mean of these 1250 comparisons was near zero (0.02±0.32) and followed an approximately normal distribution. The maximum correlation between any two trials was 0.81. The correlation values between the STAF prediction and the average response over the 50 trials therefore suggest that the prediction is capturing a large amount of the variability in the flies' responses. The trial-to-trial variability is quite large, but robust responses to stimuli are apparent over an average of a small number of trials with a small number of flies.

## DISCUSSION

We demonstrate that figure tracking and wide-field ground stabilization depend critically on the spatial tuning of the figure-tracking system (Fig. 4E). When a figure occupying merely 8% of the total visual field is localized frontally, it is tracked largely to the exclusion of wide-field motion signals impinging upon 92% of the visual field. Indeed, the spatiotemporal dynamics of the figure STAF is immune to uncorrelated wide-field motion (Fig. 4B,E, insets), thereby supporting the behavioral observation that flies readily track small figures against anti-phase ground motion (Fig. 2).

### Analyzing figure-tracking behavior: the utility of the STAF method

The motion of an object results in a distinct change in image velocity and image position on the retina. Classical experiments in flying flies sought to isolate any response to an object's position from its motion by measuring the steering response either to a flickering vertical bar held in a stationary position on the azimuth, or to a very slowly moving solid black bar on a white ground. In both cases, flies show a slow-onset, sustained response in yaw torque proportional to the azimuthal displacement of the visual figure (Pick, 1974; Poggio and Reichardt, 1976). These results had provoked criticism suggesting that because natural object tracking and fixation behaviors occur very rapidly, position coding is unlikely because of a slow position system, and is more likely to be achieved by fast motion-dependent inputs with small receptive fields tiled across the visual azimuth (Wehrhahn and Hausen, 1980). Yet, the two theories need not be mutually exclusive.

Aptekar et al. (Aptekar et al., 2012) adopted a white-noise approach to modulate the position of a distinct visual figure independently from the coherent small-field motion cues contained within it. The results showed that figure-tracking behavior is based on two spatiotemporally distinct streams of information, one (FM) that encodes primarily the DC position of a moving figure, and another that encodes the EM velocity of the small field figure surface. The EM stream transduces the space–time correlations in first-moment (mean) luminance distribution generated by the velocity of motion (and hence is akin to the traditionally defined object ‘velocity’ system). The FM stream transduces spatiotemporal disparities including flicker, second-moment luminance envelope, and higher-order features such as motion-defined motion, all of which can signal either the static position or the dynamic movement of first-order cues (and includes the traditionally defined ‘position’ system). The superposition of the two sub-systems is both sufficient and necessary to explain flies' responses in tracking higher-order figure motion signals (Aptekar et al., 2012). The STAF method also recapitulates the azimuthal variation in steering strength observed by Reichardt and Poggio (Reichardt and Poggio, 1976) [and more recently by Bahl et al. (Bahl et al., 2013)] using a very slowly revolving dark bar, which is detailed in a companion method paper (J.W.A., unpublished).

Here we go substantially further and compare STAFs for figure and ground motion to show how the composite (EM+FM) figure tracking system interacts with the wide-field ground stabilization system. Note that the trajectory of the composite figure STAF exactly matches the maximum of the EM and FM STAFs measured separately, rather than their sum [fig. S2 in Aptekar et al. (Aptekar et al., 2012)]. This demonstrates one of presumably many nonlinearities in figure processing. Yet the separate EM and FM components revealed previously compose the central features of the total figure response shown here, evidenced by the robust predictability of the method. Importantly, our method permits simultaneous measurements of both figure-tracking and optomotor responses simultaneously and removes the complication of having to estimate them independently. Flies clearly make these computations in parallel, and the interaction between the wide-field ground and figure systems is demonstrated by their capacity to fixate a 30 deg figure against a 300 deg counter-rotating ground (Fig. 2).

### Figure and ground STAFs are spatially complementary

The most striking characteristic of the STAFs constructed from the response to simultaneous figure and wide-field ground stimuli (Fig. 4A,C) is their complementary spatial profiles. The response to the figure has its highest gain when the figure is centered on the visual midline, and early in time, where and when the response to the ground motion (with a figure present) has its lowest gain. Conversely, ground response gain is high only when the figure is in the periphery. The mean of the two subsystems is a nearly flat distribution, indicating that the two STAFs are spatially complementary (Fig. 4E). This result persists for figures of increasing width (Fig. 6).

Similar prior analyses demonstrated that the figure-tracking systems operate with highest fidelity on the visual midline (Reichardt and Poggio, 1976; Aptekar et al., 2012). Insects that chase conspecifics or prey on the wing, such as blowflies, hoverflies and dragonflies, show optical specialization for improved resolution by the ommatidia of the frontal eye field (Land and Eckert, 1985; Land, 1997; Straw et al., 2006), which in part enable the remarkable target specificity of neurons within the lobula complex (Nordström, 2012) and downstream descending neurons that feed the motor centers of the thorax (Gonzalez-Bellido et al., 2013). By contrast, the eyes of *D. melanogaster* shows essentially uniform spatial acuity across the visual midline, but do have roughly 20 deg of binocular overlap corresponding with the region of maximum figure sensitivity (Heisenberg and Wolf, 1984). We might speculate that these animals possess a ‘motion fovea’ rather than an optical one, and that the figure STAF reflects that specialization.

### Figure tracking suppresses the ground optomotor response

To our knowledge, this is the first report demonstrating that the wide-field ground stabilization system is suppressed during active figure tracking, evident in the delayed onset and lower peak amplitude of the ground STAF when a figure is moving near the visual midline (Figs 4, 6, 7). Of course, this ought to be the case, or animals could never pursue objects that contrast with self-generated optic flow. One mechanism for separating the two control efforts is bandwidth fractionation in the frequency domain (Egelhaaf et al., 1988; Duistermars et al., 2007). Although our results here show that the temporal properties are indeed distinct for the two systems (Fig. 4), we have gone further to show that the spatial organization of the figure system determines whether the animal will follow the figure or the ground. We make this assertion because both the spatial and the temporal properties of the figure-tracking system are similar regardless of whether the ground is in motion (Fig. 4) (Aptekar et al., 2012). Additionally, it is the presence of a moving figure, rather than the effect of occluding input to an anisotropic ground action field, that is the key factor for ground response suppression. Our control experiments (Fig. 5) confirm that relative motion of a figure significantly interferes with the ground response early in time, and only near the visual midline, precisely overlapping with the region of maximum strength in the figure STAF.

### Possible neural mechanisms for figure–ground discrimination

Obvious candidates for the neuronal basis of figure and ground processing in flies include the giant tangential cells of the lobula plate (LPTCs), such as classes with large receptive fields sensitive to wide-field motion [e.g. the horizontal system (HS) and vertical system (VS) cells (Borst et al., 2010)]. In fruit flies, houseflies and hoverflies, high gain figure responses are also observed within HS neurons classically associated with the wide-field pathway (Reichardt et al., 1983; Schnell et al., 2010; Lee and Nordström, 2012). Some members of a loosely defined class of LPTCs show selectivity for small-field signals generated by figure motion (FD cells) (Liang et al., 2012) via inhibition generated by wide-field motion.

Genetically silencing motion-detecting inputs to this ganglion eliminates wide-field optomotor responses and strongly attenuates responses in wide-field LPTCs, but does not disrupt behavioral orientation toward a black bar on a white ground (Bahl et al., 2013). This would suggest that the lobula plate may be dispensable for figure tracking. But, the experiments by Bahl et al. defined a figure only by relative luminance, not by relative motion or higher-order flow field disparities, which can provoke behavioral figure tracking independent of the direction of figure motion (Theobald et al., 2010b), and which are robustly encoded within the HS neuron of the hoverfly lobula plate (Lee and Nordström, 2012). In addition to the lobula plate, the lobula has been shown to contain higher-order target-detecting neurons in hoverflies and dragonflies (Nordström, 2012) and fruit flies (Mu et al., 2012), and the genetic silencing of lobula projection neurons compromises behavioral sensitivity to higher-order figure motion (Zhang et al., 2013).

Whereas GABAergic inhibitory interactions between wide-field and figure-sensitive cells sculpt the receptive field of figure-detecting neurons housed in the lobula plate of larger flies (Egelhaaf et al., 1993; Warzecha et al., 1993), we have no mechanistic evidence for direct inhibition of the wide-field optomotor response by the figure system. However, it is difficult to imagine that flies could persistently fixate a figure on a counter-directional ground (Fig. 2) without some sort of synaptic suppression of wide-field processing, or how the figure motion-dependent suppression of the ground STAF (Fig. 5) occurs without direct inhibition. Indeed, figure-tracking behavior is compromised in *Drosophila* for which the vesicular GABA transporter has been mutated (Fei et al., 2010). Future experiments, perhaps with genetic reagents that manipulate GABA signaling, will be needed to directly test whether activation of the figure-tracking system is accompanied by inhibition of the wide-field tracking system, and how the spatial profile of the figure system is established.

## MATERIALS AND METHODS

### Animals and flight simulation arena

Adult female *Drosophila melanogaster*, 3–5 days post-eclosion, were reared from an iso-female colony of wild-caught flies (Card and Dickinson, 2008). Flies were cold-anesthetized, tethered to tungsten pins and placed in the center of a 32×96 pixel cylindrical green LED flight arena (Fig. 1A), as described previously (Reiser and Dickinson, 2010). An infrared LED (880 nm peak emission, Advanced Photonics, Ronkonkoma, NY, USA) illuminated the wings on an optical sensor (JFI Electronics, Chicago, IL, USA) that detected the amplitude of the left and right wingbeats. The difference in amplitude between the left and right wings (ΔWBA), as processed by this instrument, is proportional to the fly's yaw torque (Tammero et al., 2004). Data were digitized at 1000 Hz (National Instruments data acquisition PCI card, Austin, TX, USA) and recorded using MATLAB (The MathWorks, Natick, MA, USA).

### Visual stimuli

In this study, we restrict motion to the horizontal yaw plane, and thus all display contours are aligned vertically to the full extent of the flight arena display (Fig. 1A). Flies were presented with a stimulus that consisted of a vertical bar 30 deg in width, extending from −60 to 60 deg elevation. Both the ground pattern and the bar were composed of a random pattern of vertical 1 pixel (3.75 deg) stripes that were bandpass filtered to ensure that most solid bright or dark elements were 2–4 pixels (7.5–15 deg) in width and that the average contrast of both the bar and the ground was matched at 50% (i.e. half of the stripes were on and half were off; Fig. 1Bi, region of figure emphasized for illustration by reducing contrast of background in Fig. 1Bii). In this way, we ensured that the figure and background could be distinguished only by relative motion, and that there were no ‘figure-like’ elements contained within the wide-field pattern. The motion of the bar and the ground were modulated separately so that they could be controlled in open-loop feedback conditions by a prescribed function or under closed-loop feedback by the amplitude difference between the two wings. Space–time graphs illustrate the spatiotemporal correlations in luminance generated by rotating the ground or the figure (Fig. 1C).

A *prima facie* concern with this experimental design is that the figure and ground have an explicit codependence because the figure effectively masks the ground over its spatial extent: a ground motion-sensing system with marked spatial anisotropy (Krapp et al., 2001) might be disproportionately compromised by masking within its regions of highest sensitivity. To test this potential limitation of our primary experimental design, we measured the STAF using a wide-field pattern with a figure-sized gray bar occluding the random-stripe pattern (Fig. 1Biii), placed at various locations but moving according to the same m-sequence as the ground. Assuming that the ground response is mediated by elementary motion detectors that rely on spatiotemporal contrast, this gray bar reduces the coherent ground motion stimulus by the same amount that the uncorrelated figure does in our figure–ground experiment, but provides no relative motion cues. This control stimulus allows us to examine whether any influence of figure motion on the ground response, parameterized by the azimuthal position of the figure, is merely the result of masking input to the ground system in that spatial location.

### Closed-loop fixation against static and opposing ground patterns

We allowed the fly to actively control the position of a randomly striped figure moving against a randomly striped ground. We placed the bar in closed-loop control using negative gain feedback to couple the steering effort to the motion of the bar. An attempted turn to the right moved the bar to the left and vice versa. The fly's ability to stabilize the bar was tested under two conditions: first against a static ground, and second with a counter-rotating ground (Fig. 2). In this experiment, if the steering effort moved the bar 1 pixel, the ground moved 1 pixel in the opposite direction. In this case, the optomotor response to the ground motion and the bar fixation response directly oppose. Each 20 s trial was interspersed with periods in which the fly tracked a dark bar against a bright ground under negative feedback for 5 s, repeated five times (*N*=16 flies). We computed the mean vector strength, a circular statistic measuring angular dispersion, at each time point during the last 10 s of each experiment (Batschelet, 1981; Reiser and Dickinson, 2010). A vector strength equal to one indicates that all flies are fixating the figure at the same azimuth; lower vector strengths indicate a greater variance in the angle of fixation among the population of flies. We used a Rayleigh *z*-test with critical level of *P*=0.05 to assess whether the vector strength is sufficiently high to claim that flies are tracking the figure (Batschelet, 1981).

### Measuring impulse responses to figure and ground motion

In this quasilinear system (Fig. 3A), time-varying input *x*(*t*) (the position of the figure or the visual panorama) is transformed into an output *y*(*t*) (the steering response ΔWBA, proportional to yaw torque) by a filter that is characterized by a velocity impulse response (or equivalently, a positional step response) function. This impulse response may be convolved with any arbitrary input *x* to predict output *y*:
(1)
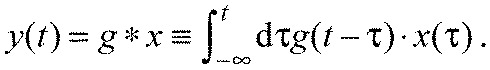

The kernel function *g*(*t*) represents the steering response to a unit step in image position, and can be measured directly by observing reactions to instantaneous pattern rotations in the display. Rotating a pattern by one display pixel (3.75 deg) evokes a steering response as a function of time that satisfies conditions of additivity, homogeneity and time invariance characteristic of a time-invariant linear system (Oppenheim et al., 1997; Theobald et al., 2010a).

Significant variations in such responses can result from different stimulus patterns, noise or drift in the fly's control system, and measurement noise in the apparatus. Thus, signal averaging from multiple repetitions is needed to construct a coherent representation of such a kernel function. Directly measuring impulse responses is time-consuming because each response must reach steady state before repeating the impulse stimulus. A vastly more efficient estimate may be obtained by stimulating the fly with *y*(*t*), a broadband white noise input *x*(*t*)=*w*(*t*), and then estimating *g*(*t*) by cross-correlation (indicated herein by the operator notation *) of the output with *w* itself (Fig. 3A). Because the expected value of the autocorrelation [w*w](*t*) of a white noise sequence is the Dirac delta function δ(*t*), and because the convolution and cross-correlation operators commute, this operation yields:
(2)
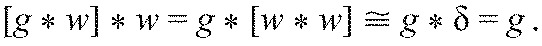

In addition, the expected cross-correlation [*w*_1_*w_2_](*t*) of two distinct realizations *w*_1_ and *w*_2_ of a white-noise sequence is zero.

Our broadband stimulus, *w*, is generated with a maximum length sequence (m-sequence), a pseudo-random sequence of unit-magnitude impulses that are used to command a series of left (−1) and right (+1) single-pixel image shifts. Although deterministic, the m-sequence approximately satisfies the autocorrelation property of a white-noise sequence, and any two distinct m-sequences of the same length approximately satisfy the cross-correlation property. The maximum excursion from the starting location varies with each m-sequence, but on average the 127-element sequences we used displaced a maximum of 5 pixels (18.75 deg). The final position of the visual stimulus is always a single pixel displaced from its start location, a convenient mathematical feature of m-sequences (Ringach and Shapley, 2004).

### Measuring impulse responses to figure and ground motion: linear filter calculation and STAF construction

To examine figure responses at each part of the visual field, we specified 24 starting positions for the figure stimulus, evenly spaced at 15 deg intervals around the full azimuth. For each trial, the figure was placed in a random starting position and the m-sequences were repeated three times for a total stimulus time of 15.6 s. As the figure moved, the starting position for each calculation of the linear filter was updated so that the final data sets include data sampled at all 96 arena positions. For each start position, flies flew two trials, one with the original figure m-sequence and one with the figure m-sequence inverted. The responses to the two were subtracted to control for any residual pattern-specific correlations. Each trial was interleaved with 5 s of active bar fixation, as described above (*N*=27 flies).

The response filters for figure motion about each azimuthal spatial position were assembled to produce a two-dimensional surface depicting the figure STAF (Fig. 4A). To measure impulse responses to the ground motion, we cross-correlated the same ΔWBA response with the ground stimulus m-sequence and constructed the ground STAF in the same way. The ground STAF is thus parameterized spatially by the position assumed by the figure during each trial. In our experiments, a steering response that is strongly correlated with the stimulus will result in a filter with large positive amplitude; a steering response that is anti-correlated with the stimulus will result in a negative-amplitude filter. Note, however, that the STAF is an incremental representation: when STAF values are negative, that does not necessarily mean that the animal's response is in the direction opposite that of image motion. Rather, it reflects the sign of the change in torque that is induced by the motion step, regardless of the sign of the prevailing total torque. Responses that are uncorrelated to the stimulus will result in a zero-amplitude filter. For clarity and to conform with prior published work (Aptekar et al., 2012), the final STAF plots were smoothed with a 4 pixel box filter. Only un-smoothed data were used for modeling (Fig. 8).

This linear analysis yields an approximation to the input–output relationship between the stimulus and the steering response that captures the first-order behavior of the system while excluding higher-order nonlinearities. The usefulness of the method for describing any particular system (and hence, for estimating the system's linearity) can be evaluated by convolving the stimulus with the calculated linear filter and comparing the resulting predictions with the measured responses. We discuss this detail of our methodology below.

### Analysis of STAF temporal dynamics

To characterize and compare the temporal dynamics of the figure and ground STAFs, we fit each filter comprising the STAFs to a model of an overdamped harmonic oscillator, plus a DC term. As has been described previously (Aptekar et al., 2012), because of the nature of the white-noise analysis employed here, and consistent with prior work using a stationary flickering bar (Pick, 1974), the DC term represents a sustained steering response (ΔWBA) to the offset position of the figure or ground. The overdamped harmonic oscillator is a simple linear model that can be described by a difference of decaying exponentials with rise and decay time constants τ_rise_ and τ_decay_, respectively, representing the onset and offset of the steering response to a velocity impulse. Additionally, to account for the physical constraints of our system, we included two fixed delays, *t*_onset_ and *t*_DC_, which represent the flies' latency to response, or reaction time, and delay to the onset of the DC position response, respectively. These latencies are implemented with the use of a differentiable approximation to a delayed step function:
(3)
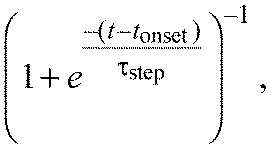

where in practice τ_step_ is set to 10 ms. In sum, our fit function was:
(4)
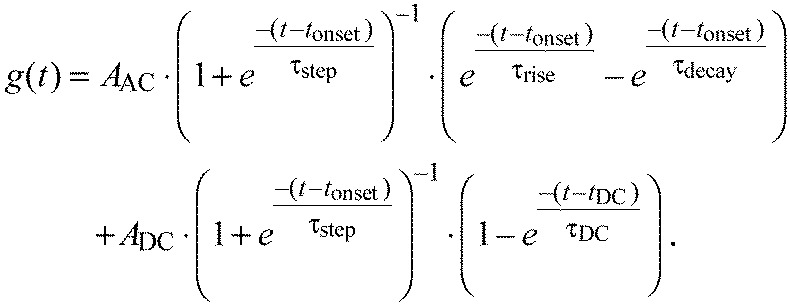



We fit the STAF data with this simple form using a nonlinear least-squares fitting algorithm with user-defined bounds for each parameter applied uniformly to all fits (custom MATLAB scripts). This method yielded fits without reaching the chosen bounds in 92% of cases across 96 azimuthal positions in both the figure and ground STAFs.

To compare the general temporal characteristics of the figure and ground STAFs, we binned the figure STAF fits from the frontal half of the arena where they are maximal – the ‘figure region’ – and the ground STAF fits from the rear half of the arena where the ground STAF is maximal – the ‘ground region’ – (Fig. 7, see green and purple bars, respectively), and compared fit parameters between these regions. A subset of these parameters that illustrates key similarities and differences in the temporal dynamics of these two systems is reported in Fig. 7C: onset delay, *t*_onset_; rise time constant, τ_rise_; offset time constant, τ_decay_; peak onset amplitude, *A*_AC_; and peak DC amplitude, *A*_DC_. Comparisons were made with unpaired *t*-tests and the *P*-values reported are Bonferroni-corrected for seven independent parameters.

### Modeling responses to figure motion against a moving background

With the same pattern used in the figure–ground discrimination STAF experiment described above (a moving randomly striped figure against a moving randomly striped background), we oscillated the bar and the background with two distinct triangle-wave position trajectories (30 deg peak-to-peak amplitude). We tested the flies' response to the figure centered at two different starting positions, one in front of the fly and one behind the fly, at each of two frequencies (0.5 and 1.2 Hz) so that the responses to figure and ground motion components would be easily distinguishable (*N*=50 trials in 10 flies). We calculated the correlation coefficient between the figure and background motion and the ΔWBA response. In doing so, we measured the amount of variation in the flies' steering effort that could be explained by figure motion and by ground motion, and thus determined the influence of each stimulus component on the overall behavior of the fly.

We next convolved the figure–ground discrimination STAFs (as calculated above) with the velocities of the figure and ground stimuli in order to predict the flies' responses to triangle-wave motion. For a given stimulus, we found the response kernel that corresponded to the azimuthal center of the triangle-wave stimulus, and convolved the stimulus trajectory with that kernel to predict the response. We then calculated the correlation coefficient between the predicted response and the flies' mean response.
